# Characterization of the surface-active exopolysaccharide produced by *Halomonas* sp TGOS-10: Understanding its role in the formation of marine oil snow

**DOI:** 10.1371/journal.pone.0299235

**Published:** 2024-05-28

**Authors:** Christina Nikolova, Gordon Morris, David Ellis, Bernard Bowler, Martin Jones, Barbara Mulloy, Tony Gutierrez

**Affiliations:** 1 Institute of Mechanical, Process, and Energy Engineering, School of Engineering and Physical Sciences, Heriot-Watt University, Edinburgh, United Kingdom; 2 Department of Chemical Sciences, School of Applied Sciences, University of Huddersfield, Huddersfield, United Kingdom; 3 Department of Chemical Sciences, School of Engineering and Physical Sciences, Heriot-Watt University, Edinburgh, United Kingdom; 4 School of Civil Engineering and Geosciences, Newcastle University, Newcastle upon Tyne, United Kingdom; 5 Institute of Pharmaceutical Sciences, King’s College London, London, United Kingdom; Texas A&M University at Galveston, UNITED STATES

## Abstract

In this study, we characterize the exopolymer produced by *Halomonas* sp. strain TGOS-10 –one of the organisms found enriched in sea surface oil slicks during the Deepwater Horizon oil spill. The polymer was produced during the early stationary phase of growth in Zobell’s 2216 marine medium amended with glucose. Chemical and proton NMR analysis showed it to be a relatively monodisperse, high-molecular-mass (6,440,000 g/mol) glycoprotein composed largely of protein (46.6% of total dry weight of polymer). The monosaccharide composition of the polymer is typical to that of other marine bacterial exopolymers which are generally rich in hexoses, with the notable exception that it contained mannose (commonly found in yeast) as a major monosaccharide. The polymer was found to act as an oil dispersant based on its ability to effectively emulsify pure and complex oils into stable oil emulsions—a function we suspect to be conferred by the high protein content and high ratio of total hydrophobic nonpolar to polar amino acids (52.7:11.2) of the polymer. The polymer’s chemical composition, which is akin to that of other marine exopolymers also having a high protein-to-carbohydrate (P/C) content, and which have been shown to effect the rapid and non-ionic aggregation of marine gels, appears indicative of effecting marine oil snow (MOS) formation. We previously reported the strain capable of utilising aromatic hydrocarbons when supplied as single carbon sources. However, here we did not detect biodegradation of these chemicals within a complex (surrogate Macondo) oil, suggesting that the observed enrichment of this organism during the Deepwater Horizon spill may be explained by factors related to substrate availability and competition within the complex and dynamic microbial communities that were continuously evolving during that spill.

## Introduction

The implications of marine microbial biopolymers in influencing the fate and degradation of petrochemical pollutants in the ocean became a topic of significant interest following the Deepwater Horizon oil spill. Deemed the worst accidental marine oil spill in the history of the oil and gas industry, the spill occurred at unprecedented depth (1,500 m below sea level) and resulted in the release of massive quantities of crude oil into the northern Gulf of Mexico. Of the estimated 4.9 million barrels (779 × 10^6^ L) of oil that spilled into the Gulf, ca. 23% (179 × 10^6^ L) of this was unaccounted for—much of this is believed to have reached the seafloor in the form of marine oil snow (MOS) [1] and in what resulted in a Marine-Oil-Snow Sedimentation and Flocculant Accumulation (MOSSFA) event [2, 3]. Subsequent calculations estimated that as much as 14% of the total oil released during the spill eventually reached the sediment in the form of MOS [4].

MOS, of macroscopic cm-size dimensions, was observed in abundant quantities on the sea surface within 2 weeks of the Deepwater Horizon blowout [5, 6] and within deep water oil plumes of the Gulf [5], and at the seafloor due to its vertical export via MOSSFA [7, 8]. Also known as oil-particle aggregates, MOS is broadly defined as floating or suspended gelatinous substances that contain droplets of oil embedded within an amorphous matrix comprised of marine biopolymers, microorganisms, mineral particles, faecal pellets and/or organic detritus [9]. Whilst the trigger(s) that lead to its formation and the mechanism(s) to how it develops into larger aggregates are not well understood, a number of studies exploring MOS composition describe exopolymeric substances—also referred to as exopolysaccharides (EPS)–are a major contributor [10–15]. However, not all types of EPS lead to forming MOS, or the quantities and size of the MOS particles can be variable depending on the source (phytoplankton- or bacterial-produced) EPS [11, 16].

Much of the EPS in the oceans is produced and extracellularly-released by microorganisms, particularly bacteria and eukaryotic phytoplankton [17, 18]. Microbial EPS has been shown to induce the formation of MOS under laboratory-controlled experiments [6, 10, 11, 16]. Considering that a significant fraction of the total dissolved organic matter (DOM) pool in the ocean water column (ca. 6.9 x 10^17^ g C) [17–19] exists as suspended EPS biopolymers (ca. 10–25% of total oceanic DOM), a strong intrusion of crude oil into the sea will likely result in the formation of MOS. Indeed, the Deepwater Horizon spill was not a rare event in this regard as evidence exists of MOS formation from several other historic oil spills [20].

Whilst our understanding of the triggers and mechanisms underlining MOS formation is still at a very early stage, various studies have shown evidence suggesting that the chemical composition, and specific chemical groups, of EPS polymers can contribute to this process, as well as in the overall oil biodegradation process. Whilst microbial biopolymers can be composed of heteropolysaccharides, lipopolysaccharides, lipoproteins, or glycoproteins, much of the pool of biopolymers in the oceans is composed of monosaccharides (*i*.*e*. EPS), some containing non-carbohydrate substituents [21]. Specific chemical groups associated with these polymers can confer them with certain functional properties, such as an ability to interface between the aqueous (seawater) environment and non-/poorly-aqueous substances such as crude oil. For example, negatively-charged moieties are commonly found on biopolymers produced by marine bacteria, such as the carboxyl groups of uronic acids associated with EPS [22, 23]. Compared to EPS produced by marine eukaryotic phytoplankton [22] and non-marine bacteria [24], EPS produced by marine bacteria generally contains higher levels of uronic acids, notably D-glucuronic and D-galacturonic acid [25]. A high uronic acids content has been shown to provide marine polymers with amphipathic properties (*i*.*e*. surface-active qualities) that allows them to interface with hydrophobic substances [26–28]. Furthermore, a large fraction of the EPS produced by bacteria in the ocean is of glycoprotein composition [19, 29]. The amino acid and peptide components found associated with these glycoprotein biopolymers have been shown to confer amphiphilic characteristics to these macromolecules [19, 26], which could explain, if at least partially, their ability to interact with oil droplets and, in turn, contribute to the formation of MOS and in microbial oil biodegradation processes.

Glycoproteins, which are protein-rich EPS, have gained considerable interest with respect to their potential role in MOS formation. These biopolymers have been shown to exhibit amphipathic properties–*i*.*e*. having both polar and non-polar chemical groups [26]–that can make up a large fraction of the EPS produced by many marine bacterial species [19, 29]. EPS enriched in hydrophobic proteins has been shown responsible for the faster and nonionic aggregation of marine gels [30–32]. Several studies have found a significant positive correlation between the relative hydrophobicity—by means of hydrophobic contact area—and the protein-to-carbohydrate (P/C) ratio of EPS from different bacterial and algal sources [see references within 33]. In general, hydrophobic interactions are a factor in microbial adhesion or ‘stickiness” and biofilm formation in aquatic environments [34–37], and may affect aggregation of other nonpolar components in the water such as oil hydrocarbons to form MOS.

These past three decades has seen an increased interest on EPS produced by members belonging to the genus *Halomonas* due to the rheological and/or surface-active properties of these biopolymers for potential commercial applications [38–41], including because they can be produced in copious quantities by some halomonads [42]. Considering the ubiquity of these organisms in the marine environment, and recognition that they are major EPS-producers, there is a significant paucity of knowledge on what ecological role these halomonad-produced polymers impart upon the marine environment and during oil spills. During the Deepwater Horizon oil spill, some of the taxa enriched on sea-surface oil slicks [43, 44] were isolated and shown in laboratory experiments to produce EPS that effected the formation of MOS in the presence of crude oil [10, 11]. Since then, *Halomonas* has commonly been reported associated with MOS particles formed in laboratory experiments using seawater collected from distinctly different regions of the same ocean, such as from the tropical to subarctic Atlantic Ocean [12, 45]. To better understand the role in the biodegradation of oil and formation of MOS during the Deepwater Horizon oil spill, in this study we investigated the chemical and physical characteristics of the extracellularly-released EPS produced by *Halomonas* sp. TGOS-10 –one of the organisms that was enriched in sea surface oil slicks of the Gulf during the Deepwater Horizon spill, and whose EPS has been shown to induce the formation of MOS [10]. We also assess the potential of the strain to degrade various aromatic hydrocarbon species in surrogate Macondo crude oil to predict its role in the biodegradation of the oil during the spill.

## Materials and methods

### Growth, maintenance and inoculum preparation of the strain

A pure culture of *Halomonas* sp. strain TGOS-10 was first isolated from a sea surface oil slick collected from the Gulf of Mexico in 2010 during the active phase of the Deepwater Horizon oil spill [10]. The strain was grown in Zobell’s marine medium 2216 (ZM/1) broth [46] and stored frozen at -80°C in 20% (v/v) glycerol. Inocula were prepared in ZM/1 broth from single colonies on ZM/1 agar plates. To assess cell growth and dynamics for production of the extracellular bioemulsifier, triplicate 2-L Erlenmeyer flasks, each containing 750 ml of ZM/1 broth supplemented with 0.5% (w/v) glucose, were inoculated with 1% (v/v) of exponentially-growing cells. The cultures were incubated at 28°C over a period of 96 hours. Two millilitre samples were taken from each flask every 24 h to monitor growth by spectrophotometry at 600 nm. Following these spectrophotometric measurements, the 2-ml samples were then used to conduct emulsification assays, as described below.

### Emulsification assay

To determine the optimal time to isolate the bioemulsifying agent from the spent medium, the emulsification activity was assessed during growth of strain TGOS-10 in ZM/1 amended with glucose (0.5% w/v). For this, after performing the spectrophotometric measurements at 600 nm, the 2 ml samples were centrifuged (13,000 xg; 10 min) to remove the cells and the resultant supernatant was then carefully taken to measure the emulsification activity using the method of [47], with some modifications. The supernatant (1.5 ml) was mixed with an equal volume of *n*-hexadecane in acid-washed (0.1 M HCl) screw-capped glass tubes (100 x 13 mm). The mixtures were manually shaken for 10 sec, then vigorously vortexed for another 10 sec, and these two steps repeated another four times. The tubes were then left to stand undisturbed for 24 h at room temperature. The Emulsification Index (EI_24_) was measured as the ratio of the height of the emulsion layer to the total original height of the mixture, multiplied by 100, and expressed as a percentage (emulsified layer) to the total original height of the mixture. The same assay was used to measure the EI_24_ of the isolated freeze-dried emulsifier (see below) when dissolved in solution and tested against *n*-hexadecane, diesel, or synthetic motor oil.

### Bioemulsifier production and extraction

The method for extraction and isolation of the emulsifying biopolymer from strain TGOS-10 was adopted from Gutierrez et al. [48]. An inoculum of the strain was prepared as described above using ZM/1 medium supplemented with glucose (final concentration 0.5% w/v). The inoculum was prepared in acid-washed glass tubes, incubated with shaking (150 rpm) at 28°C until exponential growth was reached. Growth was monitored by spectrophotometric measurement (OD_600_, optical density), and at the point when exponential growth was reached (~72 h), the culture was transferred to two 2-L Erlenmeyer flasks, each containing 750 ml of ZM/1 medium amended with 0.5% (w/v) glucose. The flasks were incubated with shaking (120 rpm) at 28°C in the dark. After 72 h, the volume of the two flasks was centrifuged at 10,000 xg for 20 min to remove the biomass. The supernatant was then filtered through 0.22 μm flow membrane to remove any residual cells. KCl to 7.5% (w/v final concentration) was dissolved into the cell-free spent medium, and then the bioemulsifying agent precipitated with the further addition of two volumes of ice-cold absolute ethanol. The mixture was left to mix for 30 min and then allowed to sit at 4°C for 24 h. The precipitated bioemulsifier was centrifuged (10,000 xg; 15 min) and then transferred into 1 kDa molecular-weight cut-off Spectra/Por^®^ Biotech cellulose ester membranes (Cole-Palmer, UK), and extensively dialyzed against distilled water over 5 days. The dialysed solution was then freeze dried and weighed to determine the yield of crude biopolymer extracted.

### Chemical analysis of the TGOS-10 biopolymer

Total amino acids content of the extracted polymer was determined by performing acid hydrolysis. For this, 3 mg of the polymer sample was dissolved in 2 ml of 6M HCl and hydrolysed for 24 h under vacuum and then dehydrated and diluted in 0.1M NCl. The total amino acids analysis was performed using a Waters 2695 Separations Module, a 2487 Dual Absorbance Detector and a 1515 Isocratic high-performance liquid chromatography pump equipped with a 300 x 3.5 mm Laborsevide 7-micron resin cation exchange column. Quantification was performed using a Sigma Acid Standard (AAS18) external standard. The total protein content was calculated from the individual amounts of amino acids.

To determine the monosaccharide composition, 2 mg of the extracted biopolymer was dissolved in 1 ml of 4 M trifluroacetic acid and hydrolysed at 121°C for 2 h [49]. The samples were then prepared for analysis by high-performance anion exchange chromatography coupled with pulsed amperometric detection (HPAEC-Pad) using a Dionex Carbopac PA-20 column on a Dionex ICS-3000 Ion Chromatography System (Dionex Corp. Sunnyvale, USA) and eluted with 0.01 M NaOH at a flow rate of 0.3 ml/min for 20 min to elute neutral sugars, and then for a further 20 min with 1 M NaOAc in 0.15 M NaOH to elute uronic acid residues. The monosaccharide composition was quantified in duplicate using external standards (fucose, rhamnose, galactosamine, arabinose, glucosamine, galactose, glucose, xylose, mannose, galacturonic acid and glucuronic acid).

The molecular weights and polydispersity determination of the TGOS-10 biopolymer were absolutely measured using an analytical system with on-line light scattering. The analysis was performed on size exclusion chromatography coupled to multi-angle light scattering and refractive index (SEC-MALS-RI) system composed from Agilent 1200 Infinity Series Analytical LC System (1200 Vacuum Degasser, 1260 Infinity Binary Pump and Auto-sampler, Agilent Technologies LDA UK Limited, Stockport, UK), connected in-line to a DAWN 8+ MALS and Optilab T-REX RI (Wyatt Technology Corporation, Santa Barbara, USA). The TSK G6000 PW (7.5 mm × 30 cm), TSK G5000 PW (7.5 mm × 30 cm) and TSK G4000 PW (7.5 mm × 30 cm) size-exclusion chromatography columns were connected in series and protected by TSK SWXL Guard column (6 mm × 4 cm) (Tosoh Bioscience, Tokyo, Japan). The columns were eluted with a 0.1 M NH4OAc + 0.05% Na3N mobile phase at flow rate of 0.6 ml/min and temperature of 30°C. The injection volume was set to 100 μl. Each sample was filtered via 0.45 μm regenerated cellulose (RC) syringe filter (17 mm, Target2^™^, Thermo Fisher Scientific, Paisley, UK) and sample concentration equalled 5 mg/ml. Before each sequence of samples, the normalization procedure was performed with a Bovine Serum Albumin standard (2 mg/ml), prepared in the same solvent. The chromatograms were recorded with Astra software v. 6.1.5 (Wyatt Technology Corporation, Santa Barbara, USA) using a refractive index increment (*dn/dc*) of 0.150 ml/g [50, 51].

### Proteolytic digestion of the biopolymer

Proteolytic digestion of the emulsifier was attempted in order to remove the protein and isolate the polysaccharide fractions to obtain clearer NMR spectra of the polysaccharide peaks. For this, 20 mg of the dried biopolymer was dissolved in 2.5 ml Proteinase K solution containing 5 mg Proteinase K (Fisher Scientific, UK) in Proteinase buffer (50 mM TrisHCl buffer pH 7.5; 1 mM CaCl_2_). The mixture was incubated at 37°C for a minimum 16 h and then placed in boiling water for 5 minutes to inactivate the Proteinase K. The solution was left to cool down and then centrifuged (10,000 xg; 10 min) to precipitate the protein fraction. The supernatant containing the polysaccharides was gently transferred into 10 kDa molecular-weight cut-off Spectra/Por^®^ Biotech cellulose ester membrane (Cole Palmer, UK) and dialysed for 2–3 days in 1 L distilled water at room temperature. The distilled water was periodically changed (4 times) during dialysis to ensure efficient removal of unwanted molecules.

### Nuclear magnetic resonance analysis

For ^1^H Nuclear Magnetic Resonance (NMR) analysis, the native extracted polymer, or the deproteinated version (see below), was dissolved in D_2_O (to 0.7 ml) containing 1 μl of 2% acetone in D_2_O as an internal reference. Spectra were acquired at 60°C on a Bruker 800MHz Neo four-channel ultra-stabilised spectrometer. One dimensional spectra were acquired using the Bruker water-suppression sequence, ‘noesygppr1d’. The number of scans was set at 32, the acquisition time was 2.03 sec, and a line-broadening factor of 0.30 Hz was applied to the data prior to processing. COSY spectra were acquired using the Bruker pulse program ‘cosygpprqf’, featuring a presaturation sequence. TD(1) was set to 2048 W and TD(2) to 512 W. TOCSY spectra were acquired using the Bruker sequence ‘dipsi2gppphzspr.3.du’ featuring a presaturation sequence—TD(1) was to 4096 W, and TD(2) to 512 W. The mixing time was 60 ms. HSQC spectra were acquired using the Bruker pulse program ‘hsqcedetgpsp.3’–TD(1) was set to 2048 W, and TD(2) to 256 W. HSQC-TOCSY spectra were acquired using the Bruker pulse program ‘hsqcdietgpsisp.2’–TD(1) was set to 2048 W, and TD(2) to 512 W. The mixing time was 100 ms.

### Hydrocarbon analysis

To determine the hydrocarbon species that the TGOS-10 strain can utilise as a sole carbon source, a synthetic seawater medium, ONR7a [52] was used and amended with surrogate Macondo crude oil (from the Marlin platform, Gulf of Mexico) as the sole carbon and energy source. For this, 250 ml of pre-autoclaved glass Schott bottles were prepared containing 45 ml ONR7a, Macondo oil to ca. 100 mg/L final concentration and inoculated with 5 ml of washed cells. The inoculum for these experiments was prepared by growing the strain in ONR7a amended with Na-pyruvate (1% w/v), then washing the cells at least three times and resuspending the cells in sterile ONR7a to 5 ml for use as inoculum. To analyse for any loss of hydrocarbons due to abiotic factors, acid-killed controls were set up in the same way, but with the exception that 85% phosphoric acid (3% final concentration) was added to bring the pH of the medium down to <1. All incubations were carried out in triplicate and incubated in parallel in the dark with gentle shaking (100 rpm) and at 21°C—a temperature similar to that *in-situ* at the sea surface in the Gulf of Mexico during the time of the Deepwater Horizon oil spill. At the termination of the experiment (day 20), all incubations were extracted for total petroleum hydrocarbons (TPH) and subsequent analysis for individual hydrocarbon constituents by gas chromatography/mass spectrometry (GC–MS).

For extraction of TPH, dichloromethane (DCM) was used at an oil/water (from the incubations) mix to DCM ratio of 2:1. The DCM fraction was removed, and the oil/water mix re-extracted an additional three times. The extracted oil sample was then diluted with DCM to ca. 5 ml and dried using anhydrous sodium sulphate. An aliquot of known volume was removed, evaporated to dryness and weighed. The gravimetric data were used to calculate the original sample weight and the weight of oil remaining.

Another known aliquot corresponding to ca. 30 mg was taken from the remaining oil sample and transferred to a 10 ml vial. An aliquot of the reference oil was weighed directly into a vial and diluted with ca. 0.3 ml DCM. Squalane and 1,1′-binaphthyl were added as standards at ca. 0.5% and 0.05% by weight of the oil, respectively. A procedural blank containing the standards was also prepared, analysed in triplicate and the reference oil was analysed in duplicate.

A chromatographic column was prepared using the sorbents silica topped with alumina. Both sorbents were pre-extracted with DCM and activated at 120°C prior to use. The sorbents were introduced as slurries in petroleum ether (b.p. range 40–60°C). The sample (sorbed to ~3 g alumina) was applied to the top of the column. The TPH fraction was eluted with 50 ml petroleum ether followed by 70 ml petroleum ether/DCM (2:5). Solvent was reduced to 3 ml using a Heidolph rotary evaporator and an aliquot was removed for gas chromatographic analysis.

The TPH fractions were analysed on a Hewlett Packard 5890 GC fitted with a split/splitless injector (300°C), a flame ionisation detector (FID) (310°C) and an HP-5 capillary column (J&W, 30 m × 0.25 mm i.d. × 0.25 μm film thickness). Samples were injected using a Hewlett Packard 6890 automatic injector. The column programme was set at 50°C for 2 min and 5°C/min to 300°C for 20 min giving a total run time of 74 min. Chromatographic data were acquired and processed using an Atlas 8.3 Chromatographic Data System (Thermo Scientific).

Analyte concentrations were measured using the peak areas of the added standards, assuming a response factor of one, and are thus semi-quantitative. Full quantitation (using a range of standards and individual analyte response factors) was not carried out since the purpose of the experiments was a comparison between different treatments, including controls, using the same analytical protocols. The aromatic hydrocarbons in the TPH fractions were analysed by GC-MS on an Agilent 7890A GC fitted with a split/split less injector at 280°C linked to an Agilent 5975C MSD, with data acquisition and processing by Agilent Chemstation software. Selected samples were analysed in full scan mode (50–600 amu/sec), but all samples were analysed in selected ion monitoring (SIM) mode using the analyte aromatic hydrocarbon molecular ions or major fragment ions. An aliquot of 1 μl of the TPH fraction diluted in hexane/dichloromethane was injected in split/splitless mode using an Agilent 7683B autosampler and the split opened after 1 min. Separation was performed on an Agilent fused silica capillary column (30 m × 0.25 mm i.d) coated with 0.25 μm 5% phenylmethylpolysiloxane (HP-5) phase. The GC was temperature programmed from 50 to 310°C at 5°C min and held at final temperature for 10 min with helium as the carrier gas (flow rate of 1 ml/min, initial inlet pressure of 50 kPa, split at 30 ml/min). Individual aromatic hydrocarbon analytes were semi-quantitatively determined by comparison of their peak areas in their respective ion chromatograms with that of the added 1,1′-binaphthyl standard (m/z 253) assuming a response factor of one.

Concentrations of hydrocarbon species/groups that were biodegraded after 20 days were calculated by subtracting the respective hydrocarbon concentrations measured in the acidified controls from those of the non-acidified incubations.

### Statistical analysis

A Student’s t-test was performed to test for significant differences (*P*<0.05) in the emulsification assay of the different hydrocarbon substrates tested, and the hydrocarbon degradation analysis against the acid-inhibited control incubations, as both these experiments were performed using triplicate tests/incubations.

## Results and discussion

### Cell growth and extracellular production of the TGOS-10 biopolymer

The growth of *Halomonas* sp. strain TGOS-10 in ZM/1 broth amended with glucose was rapid and reached the stationary phase within the first 24 h (S1 Fig). During growth in this medium, the production of the bioemulsifier was clearly correlated with intense growth, with the highest emulsification values (EI_24_ of 63% ± 2.1%) measured in the cell-free culture broth at 48 h. Thereafter, the emulsification index steadily decreased to 40% ± 1% at the end of the incubation period (96 h). However, all the emulsions that were formed when assaying the cell-free medium, and at the various sampling timepoints, remained completely stable when left to stand unshaken at room temperature for two weeks (results not shown). This indicates that the extracellularly-released bioemulsifier remained highly active, even though its concentration partially decreased (EI_24_ values as a proxy for concentration) after 48 h. Based on the highest measured emulsification index recorded at 48 h, this timepoint was selected as optimal to extract the bioemulsifier from the spent medium. For this, a fresh batch of cultures (2.25 L) was prepared and from this, 2.768 g of freeze-dried biopolymer was recovered from a total culture volume of 2.25 L, equating to 1.23 g of dried biopolymer per L of spent culture liquid.

*Halomonas* sp. strain TGOS-10 was recently found to share 100% 16S rRNA sequence identity with *Halomonas* strains TG39 [48] and MCTG39a [53], and 99% sequence identity to *Halomonas titanicae* BH1 [54]. Despite the genetic similarities and comparable growing conditions between the strains, there were, however, some major differences in emulsification and chemical composition (discussed below). From previous studies on other *Halomonas* sp., such as strain TG39 [26, 48], glucose has been shown to be a suitable carbon source for growing these organisms and producing highest quantities of their produced bioemulsifying polymer when compared to using other growth substrates, such as sucrose, mannitol or malt extract [26].

### Emulsification of petrochemicals

**Fig 1:**
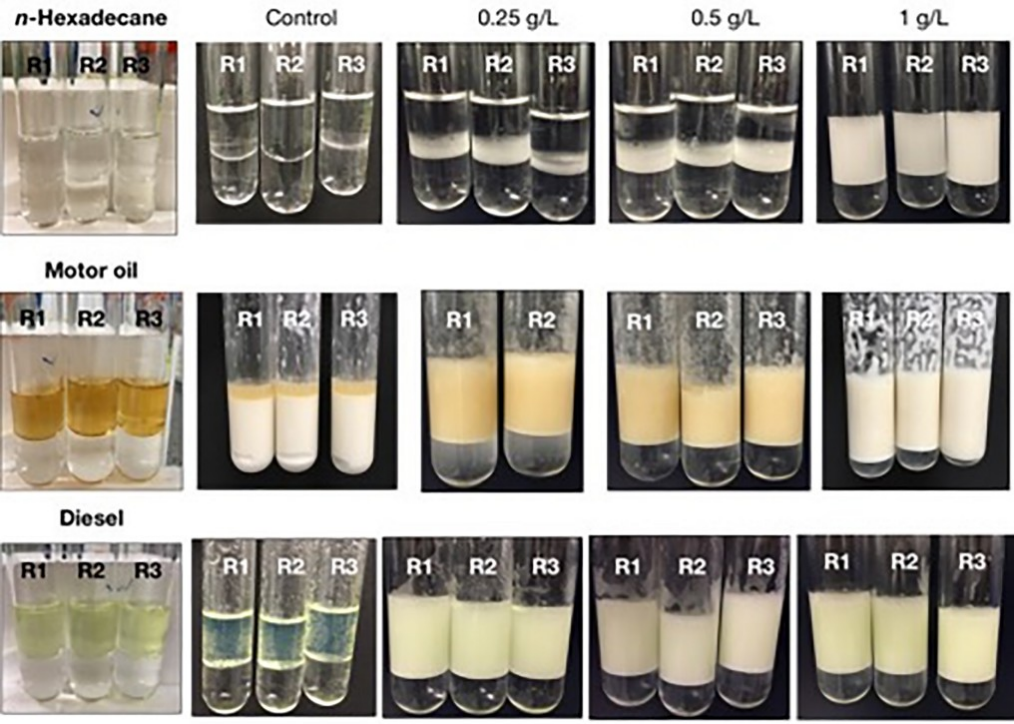
Emulsification of three different hydrocarbon compounds by *Halomonas* sp. strain TGOS-10 emulsifier (extracted and freeze-dried) in three concentrations and control (MilliQ water). Pictures on the far left show the treatments before commencing the emulsification test. The rest of the pictures show the emulsification after 24h at room temperature.

Exopolymers from various *Halomonas* sp. have been shown to emulsify individual hydrocarbon species, as well as crude oils and refined petroleum products [26, 38–42, 48, 55]. However, little is still understood on the contribution of such polymers, and those produced by members of other marine genera, in influencing the degradation of hydrocarbons during oil spills, as could be mediated by their potential ability to disperse crude oil into tiny droplets that, in turn, increases the oil’s available surface area for biodegradation by the producing organisms and other hydrocarbon-degrading bacteria. To assess the TGOS-10 exopolymer for its ability to breakdown and emulsify oil hydrocarbons, we tested this using a sole hydrocarbon (*n*-hexadecane) and two refined oils (synthetic motor oil and diesel). The polymer was dissolved in distilled and deionised water to final concentrations of 0.25, 0.5 and 1.0 g/L, each in triplicate, and the solutions used to perform the emulsification assay with the three oils. As shown in Table 1, the polymer effectively emulsified all three tested oil substrates, though emulsification values varied with polymer concentration and type of oil (Fig 1). Of the three hydrocarbon types tested, the emulsification of *n*-hexadecane was most affected by the concentration of the bioemulsifer in solution. On the other hand, the emulsification of synthetic motor oil and diesel remained relatively consistent, regardless of the bioemulsifer concentration used. At the lowest concentration used (0.25 g/L), the polymer emulsified the motor oil and diesel to values of 60–62%, while the emulsification of *n*-hexadecane was markedly less (EI_24_ of 10%) (Table 1). Doubling the polymer concentration to 0.5 g/L did not markedly increase the emulsification of any of the oils. Not unexpectedly, the highest emulsification values with all three oils occurred using the highest polymer concentration (1.0 g/L) tested, though for the diesel oil the observed increase in emulsification was not significant (*p* > 0.05). However, the emulsification of *n*-hexadecane increased significantly (by 4.5-fold; *p* < 0.05) with increasing concentrations of the bioemulsifier, from 0.5 g/L to 1.0 g/L—respectively resulting in EI_24_ values of 14% ± 3.85% to 65% ± 1.77%. For motor oil, its emulsification was also significantly increased (by 1.3-fold; *p* < 0.05) by increasing the concentration of the bioemulsifier from 0.5 g/L to 1.0 g/L. The emulsions with these oils remained stable for at least two weeks at room temperature (results not shown), demonstrating the effectiveness of the TGOS-10 polymer as a powerful emulsifier against different petrochemicals.

**Table 1:** Emulsification of a pure hydrocarbon oil (*n*-hexadecane) and two complex oils (synthetic motor oil and diesel) by the TGOS-10 bioemulsifying polymer. Emulsification was performed in distilled and deionised water with the respective oil. Emulsification activity (EI_24_) was measured after 24 hours since mixing the two phases, and is expressed as the height of the emulsion formed and expressed as a percentage of the total height of the mixture before mixing.

EPS concentration (g/L)	*n*-Hexadecane	Motor oil	Diesel
	Average % (± SD)	Average % (± SD)	Average % (± SD)
Control	0 ± 0	– ^a^	0 ± 0
0.25	10 ± 3.22	60 ± 0.51	62 ± 2.69
0.5	14 ± 3.85	64 ± 5.18	63 ± 0.64
1.0	65 ± 1.77	82 ± 5.88	64 ± 1.74

^a^ Emulsification of the synthetic motor oil Control produced what appeared to be an oil-in-water emulsion (Fig 1) that was difficult to measure and give a EI_24_ value.

These results are comparable to emulsification activities for other EPS-producing halomonads, such as *Halomonas* sp. strains TG39 and TG67 [48] when tested against *n*-hexadecane and using the same emulsification assay [47]. At concentrations as low as 0.02% (w/v), the bioemulsifiers produced by strains TG39 and TG67 produced emulsification activities of around 60%. Similarly, the TGOS-10 biopolymer at 0.025% (w/v) produced ca. 60% emulsification of synthetic motor oil and diesel oil, but not with the pure hydrocarbon *n*-hexadecane. This might suggest the potential use of the TGOS-10 bioemulsifier in applications requiring the emulsification or dispersion of complex petrochemicals.

Not surprisingly, the motor oil was emulsified in the untreated controls which contained deionised water (no bioemulsifier added), although the upper oil phase was incompletely emulsified compared to the treatments with added TGOS-10 bioemulsifier. This emulsification observed in the controls can be explained by the fact that detergent additives are commonly added to engine oils to clean and neutralise oil impurities that would otherwise cause oil deposits on essential engine parts [56]. Since the detergent additives are surfactants in themselves–*i*.*e*. having both polar (hydrophilic) and nonpolar (hydrophobic) chemical moieties—this will allow motor oil to readily mix with an aqueous phase, such as deionised water, and form stable emulsions as observed in the emulsification control tests.

### Chemical composition and molecular mass of the TGOS-10 biopolymer

The carbohydrate content of the TGOS-10 biopolymer was 1.5% of the total weight of the dried polymer. Monosaccharide analysis showed that hexoses (fucose, rhamnose, galactose and mannose), traces of amino sugars (galactosamine and glucosamine), uronic acids (galacturonic and glucuronic acid) and the pentose arabinose were present in the polymer (Table 2; S2 Fig). Galactose (72% ± 4%) and mannose (17% ± 1%) were the most abundant, while all other monosaccharides were each present at less than 3%. The monosaccharide composition of the polymer is concomitant with that of other marine bacterial exopolymers, which are generally rich in hexoses [57, 58], such as galactose which contributed 72% of the total carbohydrate composition of the TGOS-10 polymer.

**Table 2:** Monosaccharide content of the polymeric bioemulsifier from *Halomonas* sp. strain TGOS-10.

*Monosaccharide*	*Mean mol% composition* ^a^
Fucose	2 ± 0.1
Rhamnose	2 ± 0.1
Galactosamine	trace
Arabinose	2 ± 0.0
Glucosamine	trace
Galactose	72 ± 1.6
Mannose	17 ± 0.8
Galacturonic acid	trace
Glucuronic acid	3 ± 0.1
**Total (%)** ^b^	**1.5 ± 0.1**

^a^ Values are the mean of triplicate samples ± standard deviation.

^b^ Total percent values are expressed as the mean percentage of total dry weight of the polymer from triplicate determinations.

The total uronic acids content, however, was only 3%, as contributed by glucuronic acid. This is quite a low uronic acids content and which was not expected since EPS polymers produced by marine bacteria are commonly found to contain a high composition of these acids [25]. The exopolymers produced by many species of marine halomonads have been reported with a high uronic acids content [48, 53], including strain TGOS-10’s closest relative, *Halomonas* strain TG39, which produces an exopolymer containing 30% uronic acids [48]. Members of other marine genera that produce exopolymers containing a high uronic acids content include *Pseudoalteromonas* (28%) [27], *Antarctobacter* (24%) [59], and *Alteromonas* (13%) [11].

Our findings are more consistent with polymeric emulsifiers extracted from *Halomonas* species isolated from saline soils which were reported to contain relatively low levels (1–8%) of uronic acids [39, 40, 60–62]. Bacterial exopolymers used in industrial applications also have low levels of uronic acids, with the exception of gellan and bacterial alginates [63]. In addition, compositional analysis of glycolipid emulsifiers produced by *H*. *anticariensis* FP35 (also derived from saline soil) [42] and by an Antarctic psychrotrophic halomonad, *Halomonas* sp. ANT-3b [41], did not identify any uronic acids associated with these polymers. Whilst a low uronic acids content could suggest that the biopolymer produced by strain TGOS-10 might confer it with a higher solubility in water than polymers produced by other bacteria which have a much higher uronic acids content [21], a more important consideration, especially of relevance to our study, is understanding what chemical groups confer the polymer with its emulsifying properties.

The total amino acids content of the TGOS-10 biopolymer was 46.6% of the total weight of the dried polymer (Table 3), indicating that it was mainly composed of protein/peptides with a low polysaccharide content. Amino acid analysis of hydrolysed samples identified the presence of four major amino acids—aspartate, glutamate, glycine and alanine, which in total contributed 61% to the total amino acid content. The percentage contribution of polar amino acids to the total amino acid content was 13.4%, whereas that of hydrophobic non-polar amino acids was 52.7%, of charged acids 31.3%, and of amphipathic acids 2.7%. The relatively high protein content of the TGOS-10 polymer is consistent with that of other marine halomonads [10, 48, 60]. Proteins are recognised to play an essential role in the emulsifying ability of some bacterial exopolysaccharides [64, 65], including for exopolymers produced by halomonads [66, 67]. Even with some *Halomonas* sp. that produce exopolymers containing a protein component at low levels (<10% of total polymer), it has been inferred to confer those exopolymers with emulsifying qualities [42, 61, 67, 68]. We posit that the relatively high protein content of the TGOS-10 exopolymer confers a similar function, as may be effected by the ratio of total hydrophobic nonpolar to polar amino acids– 52.7:11.2 for this exopolymer. The role of amphipathic acids (2.7% of total amino acids), albeit a small percentage, will likely have contributed some effect to the bioemulsifying activity of the TGOS-10 exopolymer.

**Table 3:** Amino acid composition of the polymeric bioemulsifier from *Halomonas* sp. strain TGOS-10.

*Amino acid*	*Mean mol % composition* ^a^
Aspartate—charged	17.3 ± 0.2
Threonine—polar	6.1 ± 0.1
Serine—polar	5.1 ± 0.0
Glutamate—charged	10.1 ± 0.4
Proline—hydrophobic	5.2 ± 0.3
Glycine—hydrophobic	18.8 ± 0.8
Alanine—hydrophobic	14.8 ± 1.1
Cysteine—polar	ND
Valine—hydrophobic	4.7 ± 0.1
Methionine—amphipathic	0.7 ± 0.0
Isoleucine—hydrophobic	2.9 ± 0.0
Leucine—hydrophobic	4.5 ± 0.2
Tyrosine—amphipathic	2.0 ± 0.0
Phenylalanine—hydrophobic	1.8 ± 0.2
Histidine—polar	2.2 ± 0.2
Lysine—charged	1.9 ± 0.1
Arginine—charged	2.0 ± 0.1
**Total (%)** ^b^	**46.6 ± 1.2**

^a^Values are the mean of triplicate samples ± standard deviation; ND, not detected.

^b^Total percent values are expressed as the mean percentage of total dry weight of the polymer from triplicate determinations.

Considering that the combined protein and carbohydrate content of the exopolymer was measured to be only 48%, this means that more than 50% of the polymer was not accounted for by our chemical analysis. This refractory nature, reflected by its high resistance to acid hydrolysis conditions used in its chemical analysis, is not uncommon and has been described for other bacterial exopolysaccharides [69]. A lipid analysis on the exopolymer did not uncover any lipid associated with it (results not shown).

The SEC-MALLS-RI analysis of the TGOS-10 polymer showed it to be composed of a molecular-weight average molar mass (M_w_) of 6,440,000 ± 440,000 g/mol, with a number-average molar mass (M_n_) of 4,930,000 ± 30,000 g/mol (S3 Fig). The polydispersity index (PDI) was calculated as the ratio of weight average to number average molecular weight (M_w_/M_n_), and for the TGOS-10 polymer it was determined to be 1.31 ± 0.01. PDI is measure of the heterogeneity of sizes of molecules of a polymer in solution, and a value close to 1 indicates that the polymer is monodisperse [50].

**Fig 2:**
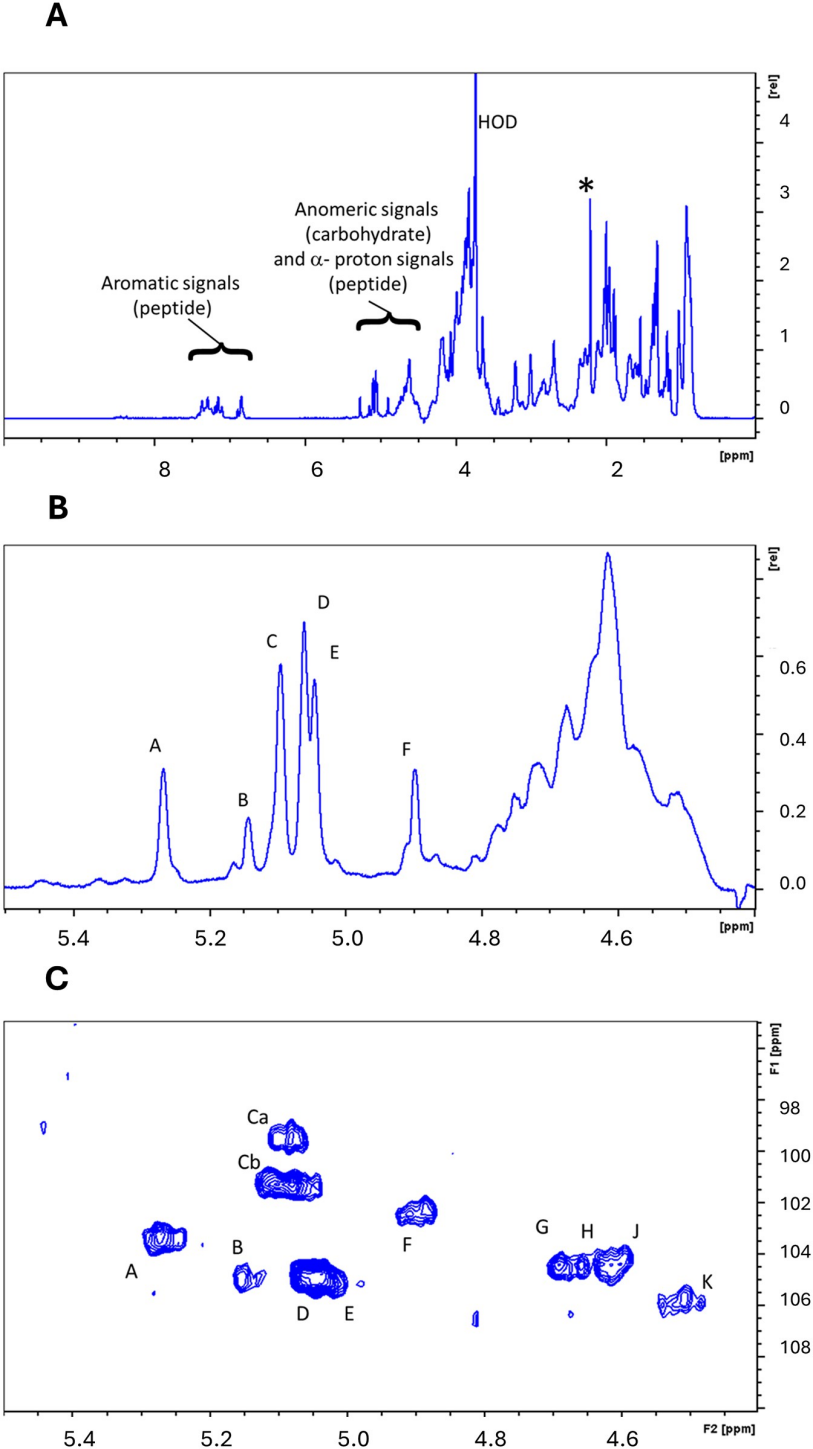
**A)** ^1^H NMR spectrum at 800 MHz, 60 ºC of the EPS (after protease treatment) in D_2_O. The spectrum includes resonances characteristic of both peptide and carbohydrate components, with well-resolved signals from aromatic amino acids between 6.5 and 7.5 ppm, and several signals between 4.9 and 5.3 ppm characteristic of a-anomeric signals from sugars. The peak marked HOD results from a small amount of H_2_O in the D_2_O NMR solvent. The asterisked peak comes from acetone, used as an internal chemical shift reference (2.218 ppm). The vertical scale is relative intensity in arbitrary units. **B)** An expansion of the ^1^H NMR spectrum in **(A)** showing the region in which signals from anomeric protons (H1) of each monosaccharide residue in the structure occur. Five clearly resolved signals in the a-anomeric region are labelled A to F. In addition, a complex envelope of signals between 4.3 and 4.8 ppm may include signals from b-anomeric protons in addition to signals from the a-protons of amino acids. **C)** An expansion of the ^1^H-^13^C HSQC NMR spectrum of the EPS (after protease treatment) to show H1-C1 cross-peaks, including those corresponding to signals A to F in the 1H spectra shown in **(A)** and **(B)**. In addition, four further cross-peaks in the ^1^H 4.7 to 4.5 ppm region, likely to come from b-anomeric positions, are labelled (G, H, J and K). These peaks could not be resolved from amino acid a-protons in the ^1^H 1D spectrum but are identified in the HSQC by their characteristic ^13^C chemical shifts.

The ^1^H NMR spectrum (Fig 2A) of the TGOS-10 polymer is complex and overlapping, containing signals attributable to both carbohydrate and peptide moieties. Signals in the aromatic region (6.5–7.5 ppm) indicate the presence of aromatic amino acids, such as tyrosine, phenylalanine and threonine. Prominent signals between 0.5 and 3.0 ppm originate largely from methylene and methyl groups in the side chains of aliphatic amino acids. Carbohydrate signals typically occur between 6.0 and 3.0 ppm, overlapping with amino acid alpha hydrogen and some beta hydrogen signals. An informative region in the spectrum lies between 6.0 ppm and about 4.4 ppm, containing signals from the anomeric (H1) protons (Fig 2B). At least six clearly resolved narrow resonances between 4.9 and 5.3 ppm are attributable to alpha-anomers, and are labelled A to F. An envelope of overlapping signals between 4.8 to 4.4 ppm may obscure further anomeric signals due to beta-anomers, among the majority of amino acid alpha hydrogens.

COSY and TOCSY spectra (not shown) revealed cross-peaks that identify the chemical shifts of H2 resonances corresponding to A-F (see S1 Table). Cross-peaks from the 4.4–4.8 ppm envelope may contain H1-H2 peaks, as well as numerous amino acid alpha hydrogen to beta hydrogen cross-peaks, including those from aspartate, glutamate/glycine, serine, threonine, alanine, valine, all identified in the amino acid analysis of this preparation.

An expansion of the heteronuclear ^1^H-^13^C HSQC spectrum is shown in Fig 2C. H1-C1 cross-peaks are visible for A-F and also for a further group of signals with ^1^H shifts between 4.8 and 4.4 ppm. This group (labelled G-K) has both ^1^H and ^13^C chemical shifts characteristic of beta-anomeric H1/C1 (and therefore distinguishable from amino acid alpha carbon/proton cross peaks with ^13^C shifts between 50 and 75 ppm). Unfortunately, 2D NMR spectroscopy (COSY, TOCSY, HSQC-TOCSY) was not able to identify any H2 or further ring proton resonances linked to signals G-K. However, low intensity cross-peaks in the HSQC-TOCSY spectrum, due to H1-C2 and C1-H2 correlations, allowed the assignment of H2 and C2 for some of the A-F series of spin systems (S1 Table).

Proteolysis using protease was intended to simplify the NMR analysis by removing peptide signals from the spectra. However, the digestion was not effective as many peptide resonances remained in the post-digestion NMR spectra. The protein content was evidently lower, but incomplete removal of protein suggests that protein bonding is quite strong and complex in the TGOS-10 exopolymer. This might also indicate protection of a peptide backbone by extensive glycosylation, rather in the manner of mucin glycoconjugates [70], but we caution that this is speculative interpretation.

Taking the NMR data together with monosaccharide analysis, residues giving rise to signals A to F may well be alpha (F may be beta) mannose, as the chemical shifts of the corresponding H2s are at relatively low field (>4.0 ppm). The signals G-K may originate from beta-sugars and are consistent with beta-galactose. The group of anomeric signals A-E bears overall similarity to the anomeric regions of the NMR spectra of mannose-containing exopolysaccharides isolated from other *Halomonas* species such as *Halomonas* sp. 2E1 [71], *Halomonas smyrnensis* [72] and *Halomonas saliphila* [73] among others. The chemical shifts of H1 and H2 of mannose in their spectra and in ours are distinctive for mannose. The similarity between our signals A to E and the same region in the published spectra leads us to suppose that the polysaccharide component of our preparation has the same type of complex mannose containing structure. Unlike our bioemulsifier preparation these exopolymers are pure polysaccharides with no protein component, and with no group of beta-anomeric signals comparable with signals G-K in our spectra. The isolation protocol for pure polysaccharides usually involves additional steps such as anion exchange chromatography [71] or proteolysis [73] after ethanol precipitation of the exopolymer. It remains unclear whether our TGOS-10 glycoprotein is covalently linked to a mannose-containing polysaccharide or whether the emulsifying agent is a mixture of separate glycoprotein and polysaccharide compounds.

### Biodegradation of the aromatic fraction by the strain

Many species of *Halomonas* have been shown to degrade aliphatic [41, 55, 74], mono-aromatic [75–79], and polycyclic aromatic [39, 40, 80–82] hydrocarbons. Because the methyl-/ethyl-naphthalenes and phenanthrenes are prevalent contaminants in the marine environment, and can significantly impact physiological processes of marine organisms [83], we mainly focused on the biodegradation of hydrocarbons with two (alkyl-naphthalenes) and three (alkyl-phenantherenes) aromatic rings in the surrogate Macondo oil by *Halomonas* sp. strain TGOS-10. At the end of the incubation (20 days), there was no significant difference (*p* >0.05) in the degradation of any of the PAHs by strain TGOS-10 compared to that in the respective acid-killed controls (S4 Fig). However, we previously reported strain TGOS-10 capable of utilising each of naphthalene and phenanthrene as a sole source of carbon and energy [44]. Taken collectively, these results suggest that the strain is unable to utilise aromatic hydrocarbons, at least those analysed in this study, when they are presented to the organism within a complex oil, or within the duration of our experiment within 20 days.

### Conclusion and ecological perspectives

In conclusion, chemical characterisation using monosaccharide, amino acid and proton NMR analyses identified the TGOS-10 exopolymer to be a glycoprotein with a high protein component. The active mechanism of steric emulsion stabilization, as shown by the very stable emulsions formed with the three oils tested, could be conferred by the proteinaceous component of the TGOS-10 exopolymer. That the proteinaceous component is important for emulsification had been shown to be the case with the plant-derived polymer gum Arabic [84], the bacterial exopolymer emulsan [85], and other microbial-derived exopolymers [86]. Since proteins can adsorb onto oil droplets and penetrate into the oil, largely mediated by hydrophobic amino acids, these points of contact act as anchoring points that stabilise the oil droplets and prevent them from coalescing and ’popping’–a mechanism that will have contributed to the natural dispersion, via emulsification, of the oil on sea surface slick during the Deepwater Horizon spill.

The relationship between hydrophobic moieties and ’stickiness’ of marine exopolymers in the formation of MOS, as well as marine snow, has not been fully elucidated. However, several published reports support the hypothesis that the formation of these aggregates is governed by ’stickiness’. In the case of MOS, this will subsequently affect the dispersion, transport and ultimate fate of the oil associated with these aggregates, where it is likely to be degraded as it falls through the water column, and further still after it settles on the seafloor via the process of MOSSFA. Particle ’stickiness’ of marine exopolymers is not only determined by the concentration of the latter in seawater, but also by their chemical composition. Exopolymers with a high protein-to-carbohydrate (P/C) content, especially those enriched with hydrophobic amino acids/peptides, have been shown to effect the rapid and non-ionic aggregation of marine gels [30–32]. Some studies reported a significant positive correlation between hydrophobicity (measured as hydrophobic contact area) and the P/C ratio of different bacterial and micro-algal exopolymers [see references within 33]. This indicates that exopolymers high in protein and enriched with hydrophobic moieties, as found for the TGOS-10 exopolymer, are likely to play importantly in aggregate formation in the ocean, including interactions that involve petrochemical spills that can result in greater dispersion/emulsification of the oil and in MOS formation. We would further note that, like with strain TGOS-10, exopolymer production by marine bacteria has been shown to follow environmental cues, such as oil spillage or application of synthetic chemical dispersants (e.g. [13]) used to treat major spills like Deepwater Horizon. Based on these observations from the literature and our results in this study, and evidence that *Halomonas* sp. TGOS-10 became enriched in sea surface oil slicks [44] and its produced exopolymer can trigger the formation of MOS [10], it is very likely that this exopolymer had contributed to the formation of the copious quantities of MOS that were observed during the Deepwater Horizon oil spill, and to the natural emulsification of the oil.

## Supporting information

S1 Fig Bioemulsifier production during growth of *Halomonas* sp. strain TGOS-10 in ZM/1 liquid medium amended with 0.5% (w/v) glucose. Emulsifying activities were derived from samples of the culture broth after removal of cells by centrifugation. Empty circles, absorbance at 600 nm; solid circles, emulsification index after 24 h (EI_24_). Each value represents the mean from three independent replicates, and error bars are standard deviation from the mean. Some error bars are smaller than the symbol.
(TIFF)

S2 Fig HPAEC-PAD chromatogram of the component monosaccharides of the exopolysaccharide produced by *Halomonas* sp TGOS-10 after hydrolysis by 4 M trifluroacetic acid at 121°C for 2 h. The peaks are from left-to-right: Fucose, Rhamnose, Galactosamine, Arabinose, Glucosamine, Galactose, Mannose, Galacturonic acid and Glucuronic acid. Chromatographic conditions are described in the Materials and Methods.
(TIFF)

S3 Fig SEC-MALS chromatogram of the exopolysaccharide produced by *Halomonas* sp. TGOS-10. The chromatogram displays the normalised light scattering (LS) at 90° angle and refractive index (RI) curves together with the molar mass of the peak calculated by MALS. Chromatographic conditions are described in the Materials and Methods.
(TIFF)

S4 Fig Concentration of aromatic hydrocarbons recovered from incubations of *Halomonas* sp. strain TGOS-10 with surrogate Macondo crude oil. Naphthalene (N), methylnaphthalenes (MN), ethylnaphthalenes (ET), dimethylnaphthalenes (DMN), phenanthrene (P), methylphenanthrenes (MP), dimethylphenanthrenes (DMP) and dibenziothiophene (DBT), methyl dibenziothiophenes (MDBT), ethyl dibenziothiophenes (ETDBT), dimethyl dibenziothiophenes (DMDBT).
(TIFF)

S1 Table Chemical shifts in ppm of H1/C1 and H2/C2 carbohydrate signals in the NMR spectrum of TGOS-10 EPS. (PDF)
