# Supplementary figures and images for: Characterization of the surface-active exopolysaccharide produced by Halomonas sp TGOS-10: Understanding its role in the formation of marine oil snow
Source: PLoS One. 2024 May 28;19(5):e0299235. doi: 10.1371/journal.pone.0299235 (PMC11132480; doi:10.1371/journal.pone.0299235)

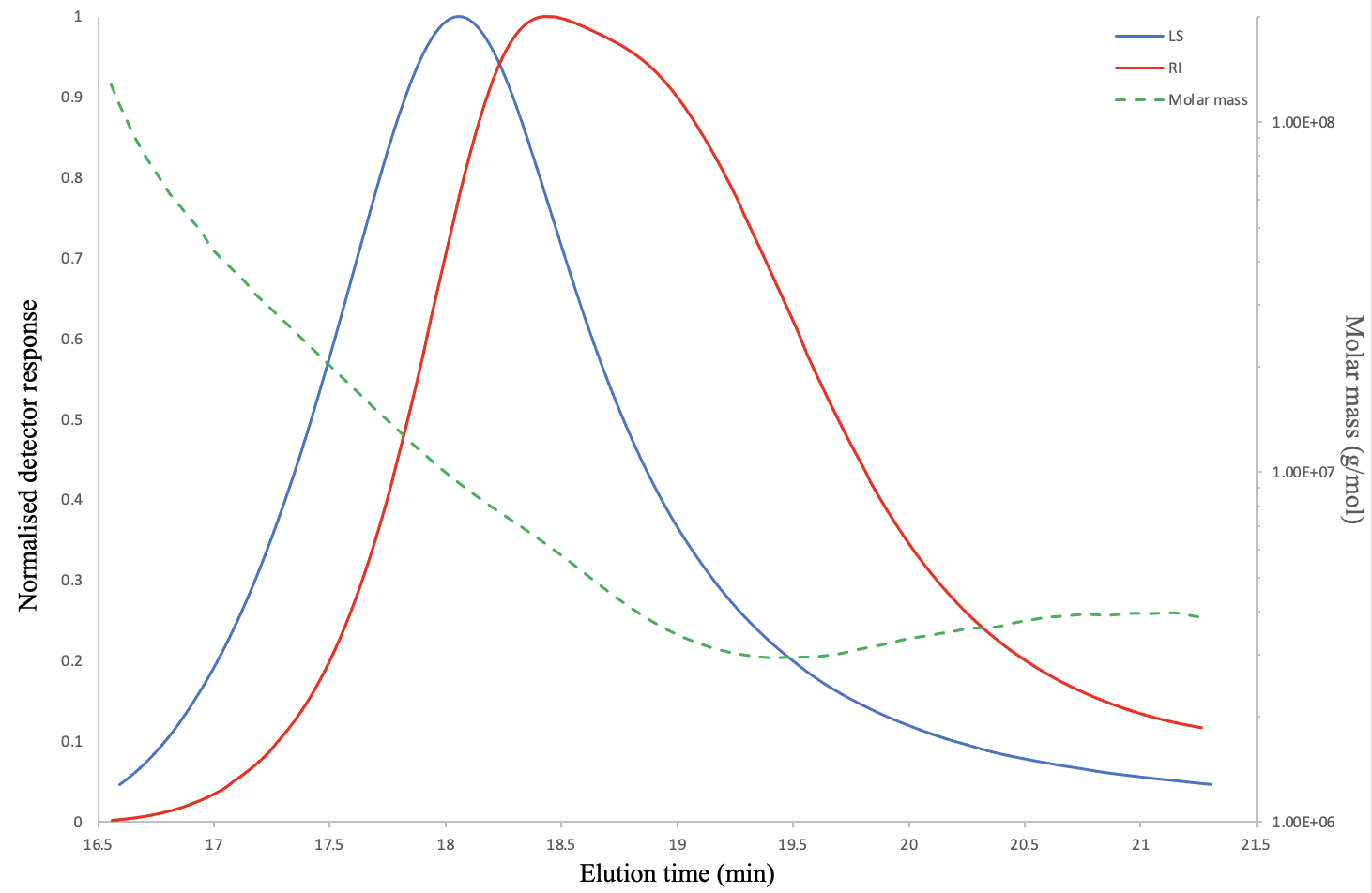

Supplement: S3 Fig — The chromatogram displays the normalised light scattering (LS) at 90° angle and refractive index (RI) curves together with the molar mass of the peak calculated by MALS. Chromatographic conditions are described in the Materials and Methods. (TIFF) [file pone.0299235.s003.tiff]

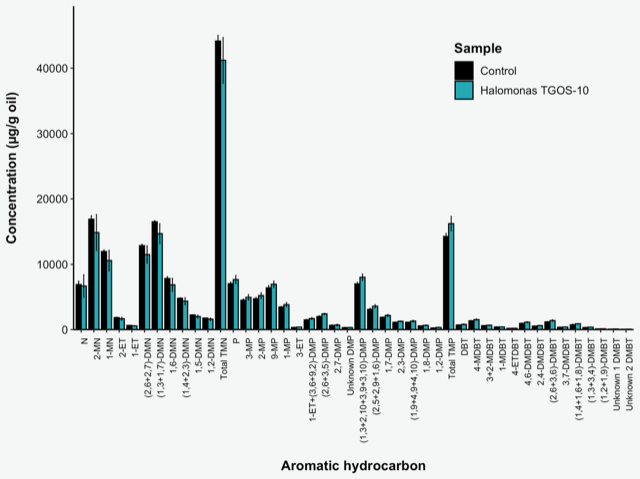

Supplement: S4 Fig — Naphthalene (N), methylnaphthalenes (MN), ethylnaphthalenes (ET), dimethylnaphthalenes (DMN), phenanthrene (P), methylphenanthrenes (MP), dimethylphenanthrenes (DMP) and dibenziothiophene (DBT), methyl dibenziothiophenes (MDBT), ethyl dibenziothiophenes (ETDBT), dimethyl dibenziothiophenes (DMDBT). (TIFF) [file pone.0299235.s004.tiff]
