# Supplementary material for: Characterization of the surface-active exopolysaccharide produced by Halomonas sp TGOS-10: Understanding its role in the formation of marine oil snow
Source: PLoS One. 2024 May 28;19(5):e0299235. doi: 10.1371/journal.pone.0299235 (PMC11132480; doi:10.1371/journal.pone.0299235)
Supplement: S1 Table — (PDF) [file pone.0299235.s005.pdf]

**Peaks**	**H1**	**C1 (HSQC)**	**H2 (COSY)**	**C2 (HSQC-TOCSY)**
A	5.27	103.3	4.12	80.9
B	5.14	104.9	4.07	72.8
C	5.10	101.2, 99.4	4.03	
D	5.06	104.8		
E	5.05	104.9	4.06	72.6
F	4.90	102.3	4.00	72.7
G	4.69	104.5	3.76	
H	4.62	104.4	3.82	
J	4.61	104.4	3.64	
K	4.50	105.6	3.35	
